# The Effect of COVID-19 Confinement in Behavioral, Psychological, and Training Patterns of Chess Players

**DOI:** 10.3389/fpsyg.2020.01812

**Published:** 2020-09-11

**Authors:** Juan Pedro Fuentes-García, María José Martínez Patiño, Santos Villafaina, Vicente Javier Clemente-Suárez

**Affiliations:** ^1^Didactic and Behavioral Analysis of Sports Research Group (ADICODE), Faculty of Sport Sciences, University of Extremadura, Cáceres, Spain; ^2^Faculty Sciences of Education and Sport, University of Vigo, Vigo, Spain; ^3^Physical Activity and Quality of Life Research Group (AFYCAV), Faculty of Sport Sciences, University of Extremadura, Cáceres, Spain; ^4^Faculty of Sport Sciences, Universidad Europea de Madrid, Madrid, Spain; ^5^Grupo de Investigación en Cultura, Educación y Sociedad, Universidad de la Costa, Barranquilla, Colombia

**Keywords:** chess, physical activity, psychological inflexibility, personality, anxiety, stress

## Abstract

The outbreak of COVID-19 has triggered a pandemic, jeopardizing global health. The sports world is also suffering enormous consequences, such as the suspension of the Olympic Games in Tokyo or, in chess, the cancelation of the World Candidates Tournament 2020. Chess is a sport characterized by high psychophysiological demands derived from long training durations, tournaments, and games, leading to mental, emotional, and physical stress. These characteristics could provide chess players a certain advantage in facing quarantine situations. This study aimed to analyze the effect of COVID-19 confinement on behavioral, psychological, and training patterns of chess players based on their gender, level of education, and level of chess played. We analyzed chess players (*N*: 450; age = 38.12 ± 14.01 years) in countries where confinement was mandatory: Professional players (*N*: 55; age = 43.35 ± 13), high-performance players (*N*: 53; age = 38.57 ± 13.46), competitive players (*N*: 284; age = 36.82 ± 13.91), and amateur players (*N*: 58; age = 39.10 ± 14.99). Results showed that chess players significantly decreased physical activity per day while increased chess practise during the confinement period. However, anxiety levels remained moderate despite the anti-stress effects of physical activity. Amateur players showed a significantly higher level of social alarm than professional and high-performance players. Moreover, professional players showed higher values of extraversion than high-performance players and amateur players. In neuroticism, professional players showed higher values than high-performance players. In addition, the professional players showed higher scores in psychological inflexibility than competitive players. Finally, chess players with the highest academic level showed higher levels of personal concern and anxiety due to COVID-19 as well as lower psychological inflexibility compared to those with a lower academic level. In conclusion, chess players, especially those with a higher academic level, might have adapted their psychological profile to fit confinement situations and the worrying levels of physical inactivity.

## Introduction

In December 2019, a novel coronavirus emerged in China, which posed an international public health emergency. This virus was named as the Severe Acute Respiratory Syndrome Coronavirus-2 (SARS-CoV-2) (Rodriguez-Morales et al., 2020). On April 6, 2020, there were 1,210,956 confirmed cases and 67,598 deaths worldwide [World Health Organization (WHO), 2020a]. Therefore, on March 11, 2020 the new coronavirus disease 2019 (COVID-19) was described as a pandemic by the World Health Organization (WHO) (2020a). With no vaccine available and no herd immunity, most of the world governments decreed a quarantine to stop the pandemic (Clemente-Suárez et al., 2020).

COVID-19 confinement produces negative psychological effects, including post-traumatic stress, confusion, or anger (Brooks et al., 2020). In this context, the confinement is the highest rated preventive measure of the Spanish population (de la Vega et al., 2020). During the confinement, most individuals are exposed to an unprecedented situation of unknown duration, being exposed to anxiety, fear, depression, or sleep disruption (Altena et al., 2020). Considering the behavioral immune system theory (Terrizzi et al., 2013), in the pandemic, people would develop these somatizations due to a negative appraisal of the situation and self-protection (Li et al., 2020). Previous studies in long confinement (105 days) produced direct modifications in stress hormone levels and immune functions (Strewe et al., 2015). Moreover, studies carried out on involuntary confinement (prisoners), showed a different incidence of psychiatric morbidity (Andersen et al., 2000).

The COVID-19 pandemic is globally affecting physical activity behaviors, forcing many people around the world to self-isolate for a prolonged time (Hammami et al., 2020). This makes it challenging to comply with the global World Health Organization (WHO) (2020b) physical activity recommendations (2020), and leads to an increase in sedentary behaviors, such as spending excessive time sat down, or using screens (playing games, watching television, using mobile devices) (Chen P. J. et al., 2020). Nevertheless, practical recommendations for staying active at home, with aerobic exercise on ergometers, bodyweight training, dance, or active video games, can help to counteract the detrimental physical and mental side effects of the COVID-19 confinement (Hammami et al., 2020).

Ahtlethes have also suffered the enormous consequences of this pandemic. For instance, the Tokyo 2020 Olympic Games was postponed until 2021 (International Olympic Committee, 2020) and the International Chess Federation (2020) (FIDE) canceled the World Candidates Tournament 2020 (FIDE, 2020). Chess is considered a sport with high psychophysiological demands where players are exposed to higher levels of stress and cognitive load (Fuentes-Garcia et al., 2018, Fuentes-Garcia et al., 2019a,b,c, 2020; Villafaina et al., 2019). Previous neuropsychological studies have shown the benefits of chess practice in executive functions, facilitating the adaptation to complex or not routine situations (Grau-Perez and Moreira, 2017; Ramos et al., 2018). Therefore, chess players showed an excellent ability for planning, self-controlling, coping, or problem-solving (Cuéllar and Díaz, 2009; Aciego et al., 2012).

Since personality modulates stress and cognition relationships (Radtke et al., 2020), a chess player’s personality could influence how they face COVID-19 confinement. In this regard, chess players are characterized for unconventional thinking and orderliness (Radtke et al., 2020), being highly competitive players, and more suspicious (Avni et al., 1987), and introverted (Vollstadt-Klein et al., 2010) than non-players. Interestingly, personality differences are evident even in young children who play chess. Children, who scored high in intellect/openness and energy/extraversion, are more likely to play chess, while children who score higher on agreeableness are less likely to play chess (Bilalic et al., 2007). Then, this study aimed to analyze the effect of COVID-19 confinement in behavioral, psychological, and training patterns of chess players based on their gender, level of education, and level of chess game.

## Materials and Methods

### Participants

A total of 450 chess players (38.12 ± 14.01), residents in 29 different countries of Asia, America, Africa, and Europe were analyzed. All the participants competed in the World Chess Federation (FIDE) and were classified according to the ranking system developed by Elo (1978). They were divided into four groups: (1) Professional players: players holding the highest level qualification or the second-highest level awarded by the FIDE, Grand Master and International Master (*N*: 55; age = 43.35 ± 13; ELO = 2414.15 ± 157); (2) High-performance players: players holding the third or fourth highest level of FIDE, FIDE Master and Master Candidate (*N*: 53; age = 38.57 ± 13.46; ELO = 2096.55 ± 156.49); (3) Competitive players: players with FIDE ranking (*N*: 284; age = 36.82 ± 13.91; ELO = 1743.34 ± 276.52) and (4) Amateur players: people who practice chess regularly but do not compete in FIDE tournaments (*N*: 58; age = 39.10 ± 14.99).

The inclusion criteria were: (a) be a chess player of 18 years or older, (b) live, at the time of data collection, in a country where COVID-19 confinement was decreed, (c) have read and signed the written informed consent.

Before participation, experimental procedures were explained to all the participants who gave their voluntary written informed consent, following the Declaration of Helsinki. All the procedures were approved by the Commission of Bioethics and Biosecurity of the University of Extremadura (Spain) (approval number: 57/2020).

### Procedure

Chess players completed an online-based questionnaire between March 3, 2020 and April 14, 2020. Firstly, they had to sign the informed consent and then disclose the following information:

#### Personal Information

• Age, gender, and current country of residence.

#### Academic and Sport-Related Information

• Academic training (university training, professional training, and high school).
• Indicate the current ELO FIDE, if appropriate.
• Highest FIDE qualification (Grand Master, International Master, FIDE Master, or Master Candidate).
• Before the COVID-19 confinement: how long did you practice chess (playing, giving or receiving classes) approximately daily, on average, considering the 7 days of the week (Nothing, Less than 30 min, Between 30 min and 1 h, between 2 and 3 h, between 4 and 5 h, between 6 and 7 h, 8 or more h)?
• In the present moment (during the confinement): how long did you practice chess (playing, giving or receiving classes) approximately daily, on average, considering the 7 days of the week (Nothing, Less than 30 min, Between 30 min and 1 h, between 2 and 3 h, between 4 and 5 h, between 6 and 7 h, 8 or more h)?
• Before the COVID-19 confinement: how long did you do physical activity (sports, gymnastics) approximately daily, on average, considering the 7 days of the week (Nothing, Less than 30 min, Between 30 min and 1 h, between 2 and 3 h, 4 or more h)?
• In the present moment (during the confinement): how long did you do physical activity (sports, gymnastics) approximately daily, on average, considering the 7 days of the week (Nothing, Less than 30 min, Between 30 min and 1 h, between 2 and 3 h, 4 or more h)?

#### Individual Perceptions About COVID-19 Crisis in a Liker 1–5 Scale (Adapted From de la Vega et al., 2020)

• Because of the current confinement rules, I consider my options to get my best performance in the most important chess competitions when they are already allowed.
• In this context of COVID-19, I am satisfied with the level of support that public institutions have had with chess players to try to maintain the highest level of preparation.
• Level of personal concern about COVID-19.
• Perception of social alarm by COVID-19.
• Control perception level to avoid getting infected by COVID-19.
• Level of personal care to avoid contagion by COVID-19.
• I consider that the current situation generated by COVID-19 has dramatically affected my chess training routines.

#### Psychological Profile

• Personality was assessed by the brief version of the Big Five Personality Inventory, where extraversion, agreeableness, conscientiousness, neuroticism, and openness to experience factors were analyzed (Rammstedt and John, 2007).
• Loneliness was evaluated by the short version of the UCLA Loneliness Scale (Hughes et al., 2004).
• Psychological inflexibility was measured by the Acceptance and Action Questionnaire-II. It is a 7-item questionnaire where participants must respond in a 1–7 scale (Ruiz et al., 2013).
• Anxiety was assessed by the State-Trait Anxiety Inventory (STAI) short form (Marteau and Bekker, 1992).

### Statistical Analysis

Based on the results of Kolmogorov-Smirnov and Shapiro-Wilk tests, non-parametric tests were used.

The Chi-Square tests were performed (χ^2^) to analyze the ordinal categorical variables related to the number of chess and physical activity practises. Mann-Whitney *U* tests were conducted to investigate gender-based differences in ELO and psychological variables. Moreover, Kruskal-Wallis tests were performed to investigate between-group differences (according to the chess performance and level of study) in the psychological variables. The Mann-Whitney *U*-test with the Bonferroni correction for multiple comparisons was conducted to explore pairwise differences.

Kendall’s Tau b (τb) was used to explore the correlation between the practice of physical activity and the practice of chess as well as the psychological profile of chess players.

Effect sizes (r), for each test, were calculated. It is classified as follows: 0.5 is a large effect, 0.3 is a medium effect and 0.1 is a small effect (Fritz et al., 2012).

## Results

Table 1 showed the descriptive data such as the number of subjects (*N*), Mean (*M*) and Standard deviation (*SD*) of the age, ELO FIDE, and the different variables associated with the Individual perceptions about COVID-19 and the psychological profile. The recruited data (*N* = 450), were grouped by age (*M* = 38.12; *SD* = 14.01), gender [*N*_male_ = 400 (88.9%), *N*_Female_ = 50 (11.1%)], level of education [*N*_Universitary_ = 284 (63.1%), *N*_Professional_ = 72 (16%), *N*_high school_ = 94 (20.9%)], and game level according to FIDE [*N*_Professional players_ = 55 (12.2%), *N*_High–performance players_ = 53 (11.8%), *N*_Competitive players_ = 284 (63.1%), *N*_Amateur players_ = 58 (12.9%)].

**TABLE 1:** Descriptive statistics of the study variables.

	*N*	*M*	*SD*
Age	450	38.31	13.70
ELO FIDE	392	1885.21	349.33
Personal concern	450	3.68	1.15
Perception of social alarm	450	4.06	0.98
Perception of control to avoid contagion	450	4.15	0.85
Personal care to avoid contagion	450	4.01	0.95
Altered options for maximum chess performance	450	2.82	1.28
Satisfaction with institutional support	450	3.00	1.26
Alteration of chess training routines by COVID	450	4.59	0.80
Extraversion	450	5.17	1.79
Agreeableness	450	5.58	1.35
Conscientiousness	450	6.78	2.09
Neuroticism	450	4.87	1.87
Openness to experience	450	7.67	1.78
Loneliness (UCLA)	450	4.86	1.77
Psychological inflexibility	450	15.00	6.35
Anxiety	450	12.26	3.54

The Chi-Square tests showed significant higher scores in physical activity practice before confinement (χ^2^ = 186.71 *p* < 0.001 τb = 0.32). Significant effects were observed in the four groups (professional players, high-performance players, competitive players, and amateur chess players) (see Table 2).

**TABLE 2:** Physical activity practise before and during the confinement.

Physical activity practice	Professional players *N* (%)		High-performance players *N* (%)		Competitive players *N* (%)		Amateur players *N* (%)	
	Before	During	Before	During	Before	During	Before	During
Did not practise PA	7 (12.7%)	16 (29.1%)	5 (9.4%)	16 (30.2%)	47 (16.5%)	80 (28.2%)	12 (20.7%)	23 (39.7%)
Less than 30 min/day	9 (16.4%)	17 (30.9%)	16 (30.2%)	12 (22.6%)	61 (21.5%)	89 (31.3%)	8 (13.8%)	10 (17.2%)
Between 30 and 60 min/day	29 (52.7%)	18 (32.7%)	17 (32.1%)	20 (37.7%)	116 (40.8%)	91 (32%)	23 (39.7%)	15 (25.9%)
Between 2 and 3 h/day	7 (12.7%)	2 (3.6%)	11 (20.8%)	2 (3.8%)	44 (15.5%)	19 (6.7%)	8 (13.8%)	5 (8.6%)
More than 4 h/day	3 (5.5%)	2 (3.6%)	4 (7.5%)	3 (5.7%)	16 (5.6%)	5 (1.8%)	7 (12.1%)	5 (8.6%)
	**Chi-squared**	***p*-value**	**Chi-squared**	***p*-value**	**Chi-squared**	***p*-value**	**Chi-squared**	***p*-value**
	37.758	0.002	28.955	0.024	106.056	< 0.001	34.368	0.005

*PA, Physical activity; min, minutes; h, hours.*

Regarding chess practice (before and during confinement) results showed significantly higher scores in practice time during the COVID-19 confinement (χ^2^ = 367.68 *p* < 0.001 τb = 0.12). Only amateur chess players did not significantly change the chess practice during confinement (Table 3).

**TABLE 3:** Chess practise before and during the confinement.

Chess practise	Professional players *N* (%)		High-performance players *N* (%)		Competitive players *N* (%)		Amateur players *N* (%)	
	Before	During	Before	During	Before	During	Before	During
Did not play chess	1 (1.8%)	2 (3.6%)	3 (5.7%)	3 (5.7%)	7 (2.5%)	5 (1.8%)	1 (1.7%)	2 (3.4%)
Less than 30 minutes/day	4 (4.7%)	5 (9.1%)	4 (7.5%)	7 (13.2%)	47 (16.5%)	22 (7.7%)	10 (17.2%)	4 (6.9%)
Between 30 and 60 min/day	9 (16.4%)	6 (10.9%)	18 (34%)	9 (17%)	105 (37%)	63 (22.2%)	22 (37.9%)	19 (32.8%)
Between 2 and 3 h/day	9 (16.4%)	10 (18.2%)	17 (32.1%)	16 (30.2%)	67 (23.6%)	99 (34.9%)	13 (22.4%)	16 (27.6%)
Between 4 and 5 h/day	12 (21.8%)	14 (25.5%)	5 (9.4%)	9 (17%)	28 (9.9%)	47 (16.5%)	7 (12.1%)	11 (19%)
Between 6 and 7 h/day	13 (23.6%)	8 (14.5%)	2 (3.8%)	1 (1.9%)	16 (5.6%)	22 (7.7%)	3 (5.2%)	3 (5.2%)
More than 8 h/day	7 (12.7%)	10 (18.2%)	4 (7.5%)	8 (15.1%)	14 (4.9%)	26 (9.2%)	2 (3.4%)	3 (5.2%)
	**Chi-squared**	***p*-value**	**Chi-squared**	***p*-value**	**Chi-squared**	***p*-value**	**Chi-squared**	***p*-value**
	71.573	< 0.001	55.368	0.021	307.057	< 0.001	39.425	0.319

*PA, Physical activity; min, minutes; h, hours.*

Mann Whitney U showed a significantly higher ELO level [*Z* = -3.156, *p* < 0.002, effect size (*r*) = 0.15] in men when compared to women. Moreover, the ELO FIDE score [*Z* = -2.084, *p* < 0.037, effect size (*r*) = 0.10] was higher in men. Regarding the differences in the perception they have about COVID-19, only differences in the level of personal care to avoid infection appeared, with higher scores in the female group [*Z* = -2.474, *p* < 0.013, effect size (*r*) = 0.12]. Finally, in the rest of the variables studied, anxiety and personality, only significant differences in neuroticism were found with higher scores in the female group [*Z* = -1.982 *p* < 0.047, effect size (*r*) = 0.93].

Kruskal-Wallis tests showed significant differences in the perception of social alarm, extraversion, neuroticism, and cognitive inflexibility (*p* < 0.05). Pairwise comparisons showed that differences were observed between professional players and amateur players, with higher values in the professional players [*Z*_(4,1)_ = 61.875 *p* < 0.007, effect size (*r*) = 0.29]. Differences were also observed between the high-performance players and the amateur players, with higher scores in the high performance group [*Z*_(4,2)_ = 66.154 *p* < 0.005, effect size (*r*) = 0.31]. Regarding personality variables, differences in extraversion were found between the amateur players and the high-performance players, with higher scores in the high performance group [*Z*_(4,2)_ = 76.791 *p* < 0.002, effect size (*r*) = 0.36]. In neuroticism, statistically significant differences were obtained between the professional players and the high-performance players, with higher scores in the high performance group [*Z*_(1, 2)_ = 78.052 *p* < 0.002, effect size (*r*) = 0.37]. Lastly, competitive players showed greater psychological inflexibility than professional players [*Z*_(1,3)_ = 50.504 *p* < 0.008, effect size (*r*) = 0.23].

The Kruskal-Wallis analyses also showed differences when comparing chess players according to the level of study. Thus, chess players with a high school education showed higher personal concern than those with university studies [*Z*_(1, 3)_ = 56.639 *p* < 0.001, effect size (*r*) = 0.26], as well as those with professional training showed higher scores than those with a high school level education [*Z*_(2,3)_ = 67.747 *p* < 0.003, effect size (*r*) = 0.31]. Regarding cognitive inflexibility, chess players with professional training showed higher values than those with university training [*Z*_(1,2)_ = 44.976 *p* < 0.026, effect size (*r*) = 0.21]. Regarding anxiety level, chess players with a high school level education showed significantly higher values than those with university [*Z*_(1,3)_ = 51.045 *p* < 0.003, effect size (*r*) = 0.24] and professional training [*Z*_(2,3)_ = 61.166 *p* < 0.008, effect size (*r*) = 0.28].

Significant positive correlations between chess practice and physical activity both before (*p* < 0.005 τb = 0.102) and during confinement (*p* < 0.005 τb = 0.089) were found. In addition, significant correlations were found between different variables in the psychological profiles of chess players (see Table 4 for further details).

**TABLE 4:** Correlational analysis of study variables.

	1	2	3	4	5	6	7	8	9	10	11	12	13	14	15
1. Concern	1														
2. Alarm	0.22**	1													
3. Control	0.22**	0.10**	1												
4. Personal care	0.35**	0.13**	0.44**	1											
5. Influence Performance	−0.07*	–0.07	0.07	0.00	1										
6. Institutions support	−0.07*	–0.01	0.08	0.01	0.19**	1									
7. Training routines	0.09*	0.07	0.01	0.06	0.01	–0.07	1								
8. Extraversion	–0.01	0.12**	–0.02	–0.03	–0.01	0.00	–0.03	1							
9. Agreeableness	0.02	–0.03	–0.03	–0.06	–0.07	0.03	0.03	−0.09*	1						
10. Conscientiousness	0.10**	0.04	0.13**	0.18**	0.08*	–0.01	0.06	–0.02	–0.02	1					
11. Neuroticism	0.10**	0.01	−0.08*	–0.04	–0.06	0.00	0.05	–0.05	0.00	−0.09**	1				
12. Openness	0.06	–0.04	0.03	0.12**	0.05	–0.03	0.04	−0.48**	–0.06	0.13**	0.00	1			
13. Loneliness	0.03	–0.01	–0.02	–0.06	−0.07*	–0.06	0.07	–0.05	0.08*	−0.13**	0.24**	−0.07*	1		
14. Psychological inflexibility	0.07*	–0.02	–0.03	−0.11**	−0.08**	0.00	0.05	–0.07	0.06	−0.19**	0.29**	–0.06	0.38**	1	
15. Anxiety	0.28**	0.05	–0.05	0.00	−0.12**	−0.09**	0.17**	0.00	0.06	–0.06	0.31**	–0.03	0.17**	0.28**	1
M	3.68	4.06	4.01	4.26	5.84	2.40	3.09	5.17	5.58	6.78	4.87	7.67	4.86	15.00	12.26
SD	1.16	0.99	0.96	0.80	2.55	1.67	1.38	1.79	1.35	2.09	1.88	1.78	1.77	6.36	3.54
N	450	450	450	450	450	450	450	450	450	450	450	450	450	450	450

*P ≤ 0.05; **P ≤ 0.01.

## Discussion

This research aimed to analyze the effect of COVID-19 confinement in behavioral, psychological, and training patterns of chess players, based on their gender, level of education, and level of chess game. The study showed that chess players significantly decreased physical activity per day while increased chess practise during the confinement period. However, anxiety levels remained moderate despite the anti-stress effects of physical activity.

In the present confinement context, increased somatization of anxiety, resulting from the perception of lack of control in adapting to contextual demands (Halabchi et al., 2020) and the imposition of a restriction of liberty or perception of non-voluntary self-isolation was described (Halabchi et al., 2020). Its health impact may be related to the duration of the confinement (extended periods are associated with poorer mental health, avoidance behaviors, and anger), the fear of infection, frustration, and boredom, inadequate supplies (e.g., water, clothes, accommodation), or inadequate information (Brooks et al., 2020). However, the anxiety values evaluated could be considered as medium, despite the low values of physical activity during the pandemic, since it has an anxiolytic effect (Petzold et al., 2020). These results could be related to the higher cognitive resources and wide experience of these high-performance athletes when coping with anxiety contexts such as competitions (Belinchon-deMiguel et al., 2019).

Interestingly, 15.8% of chess players did not practise physical activity (sports, maintenance gymnastics) before the confinement (20.9% less than 30 min on average a day) and this percentage went to almost double, 30% (28.4% less than 30 min on average a day) during the confinement. The 12.7% (29.1% during confinement) of professional chess players did not carry out any type of physical training before COVID-19, and 16.4% (30.9% during confinement) did less than 30 min per day. This is a controversial fact since a good physical condition is recommended to obtain the maximum chess performance (Alifirov et al., 2017). The physical activity analyzed did not accomplish the health requirements of World Health Organization (WHO) (2020c), which is in line with the increased sedentarism of the general population around the world (Middelbeek and Breda, 2013).

Focusing on gender-based differences, the results of the present study showed a significantly higher ELO FIDE in men than in women. This finding is in line with a previous study (Chess-rankings, 2020), and could be explained by different factors such as participation rates, degree of involvement, starting age, and perseverance. Our results also showed that women reported a higher level of care to avoid infection than men. This is consistent with a previous study (de la Vega et al., 2020) in the Spanish population where men and women significantly differed in compliance with safety measures, exercising proper care to wash hands, and in keeping at least 1.5 m distance from others in public spaces. However, this greater compliance with safety measures does not translate into a higher number of infections (Wenham et al., 2020). Nevertheless, there seems to be higher mortality and vulnerability to the disease in men (Epidemiology Working Group for Ncip Epidemic Response and Chinese Center for Disease Control and Prevention, 2020) which could be due to differences in the immunological response (Chen N. S. et al., 2020) or the prevalence of smoking (Liu et al., 2017).

Regarding chess performance groups, professional and high-performance groups reported higher values of social alarm than amateur ones. This may be due to a high control perception to avoid infection and personal care to avoid infection, which is also shown in our results. In addition, positive correlations were found between the level of personal concern, the perception of social alarm, the control perception level to avoid getting infected, and the level of personal care to avoid infection by COVID-19. Similar results were obtained in a previous study (de la Vega et al., 2020) regarding attitudes toward COVID-19 in Spain. Moreover, Vollstadt-Klein et al. (2010) showed that elite chess players showed a direct correlation between skill and extraversion, which is in line with an effect of extraversion on the psychological and physical strain. These results are in line with ours since our participants showed that the highest level of performance had the highest values of extraversion and neuroticism. In contrast, in the study of Vollstadt-Klein et al. (2010), the authors did not find significant differences between the level of neuroticism between competitive players and non-players.

Differences in cognitive inflexibility were not found between the different performance groups. Although previous studies as Grau-Perez and Moreira (2017) or Ramos et al. (2018), showed that children who practice chess have higher scores on tasks that value cognitive flexibility than those who do not. This is probably because chess practice facilitates adaptation to complex or novel situations, which are not routine and demand control mechanisms to resolve effectively. However, when participants were divided by education levels, the professional education level group showed higher values than the university level group. These results are in line with a previous study showing a negative correlation between educational level and psychological inflexibility (Wicksell et al., 2010). On the other hand, the psychological inflexibility was related to an inadequate adaptive response in the confinement situation, since it correlated with a negative perception in their sports performance and more neuroticism trait, loneliness feeling, and anxiety. In this sense, psychological inflexibility has been shown to be detrimental to mental health (Makriyianis et al., 2019).

The present study has some limitation that should be addressed. Firstly, the use of non-validated questionnaires for assessing some of the outcomes. Second, due to COVID-19 confinement, only self-reported answers were possible to obtain. Therefore, physical activity data should be taken with caution. Thus, future studies should validate these questionnaires and use direct methods to assess physical activity, such as objective monitoring devices (accelerometers and pedometers).

## Conclusion

Chess players perceived that COVID-19 confinement negatively affected their physical activity profile, increasing chess practice, despite anxiety levels remained moderate. The perception of alarm is higher in the lower level of play, while the extraversion, neuroticism, and psychological inflexibility is higher in the higher level of play. A higher academic level seems to be related to higher levels of personal concern and anxiety due to COVID-19 and lower psychological inflexibility levels. Chess players, especially those with a higher chess level, might have an adapted psychological profile to confinement situations, as well as worrying levels of physical inactivity.

## Data Availability Statement

The raw data supporting the conclusions of this article will be made available by the authors, without undue reservation, to any qualified researcher.

## Ethics Statement

The studies involving human participants were reviewed and approved by the Commission of Bioethics and Biosecurity of the University of Extremadura (Approval No. 57/2020). The patients/participants provided their written informed consent to participate in this study.

## Author Contributions

JF-G and MM conceived the study and collected the data. JF-G and VC-S designed the questionnaire and analyzed the data. JF-G and SV designed the tables. JF-G wrote the manuscript. JF-G, MM, SV, and VC-S provided critical revisions on the successive drafts. All authors approved the manuscript in its final form.

## Conflict of Interest

The authors declare that the research was conducted in the absence of any commercial or financial relationships that could be construed as a potential conflict of interest.
